# Retraction: By up-regulating μ- and δ-opioid receptors, neuron-restrictive silencer factor knockdown promotes neurological recovery after ischemia

**DOI:** 10.18632/oncotarget.28712

**Published:** 2025-04-04

**Authors:** Hui-Min Liang, Li-Jiao Geng, Xiao-Yan Shi, Chao-Gang Zhang, Shu-Yan Wang, Guang-Ming Zhang

**Affiliations:** ^1^Department of Neurology, Huaihe Hospital of Henan University, Kaifeng 475000, China; ^2^Institute of Traditional Chinese Medicine, Henan University, Kaifeng 475000, China; ^3^Department of Anesthesiology, Tongren Hospital, Shanghai Jiao Tong University School of Medicine, Shanghai 200336, China; ^*^These authors contributed equally to this work

**This article has been retracted:** Oncotarget has completed its investigation into the image duplications within this paper. It was found that in Figure 3A, the BrdU immunohistochemistry staining images of brain tissues from the ‘sham’ and ‘NRSF’ groups contain overlaps with panels B and C in Figure 5 of a previously published unrelated paper [1], which has since been retracted. The “normal” group image also overlaps with the image in Figure 4A of a concurrently published unrelated paper [2]. Additionally, the Western blot b-actin image in Figure 7A overlaps with the b-actin image in Figure 3B of the same paper [2]. Moreover, immunostaining and Western blot images were also reproduced in numerous later publications.

Despite multiple attempts to contact the authors regarding these concerns, we have not received a response. Therefore, the Editorial decision was made to retract this paper.

Original article: Oncotarget. 2017; 8:101012–101025. 101012-101025. https://doi.org/10.18632/oncotarget.18195
